# Herbivory on Banker Plants Enhances Resistance-Related Responses of Neighboring Tomato Plants to the Two-Spotted Spider Mite

**DOI:** 10.3390/plants15040665

**Published:** 2026-02-22

**Authors:** Tomoya Tasaki, Yuka Okemoto, Karin Nakamura, Norihide Hinomoto, Masayoshi Uefune

**Affiliations:** 1 Department of Agrobiological Resources, Faculty of Agriculture, Meijo University, Nagoya 468-8502, Japanmuefune@meijo-u.ac.jp (M.U.); 2 Laboratory of Ecological Information, Graduate School of Agriculture, Kyoto University, Kyoto 606-8502, Japan; hinomoto.norihide.8m@kyoto-u.ac.jp

**Keywords:** *Nesidiocoris tenuis*, zoophytophagous, plant resistance, banker plants

## Abstract

Banker plants are non-crop plants that sustain populations of biological control agents prior to pest outbreaks, offering a preventive strategy within integrated pest management (IPM). Their benefits have primarily been attributed to top-down regulation via natural enemy-mediated pest suppression; however, their potential bottom-up effects remain largely unexplored. Here, we show that airborne cues emitted from banker plants infested with the zoophytophagous mirid bug *Nesidiocoris tenuis* altered the performance of the two-spotted spider mite *Tetranychus urticae* on neighboring tomato plants *Solanum lycopersicum*. Exposure to airborne cues from infested sesame *Sesamum indicum* significantly reduced mite fecundity, whereas those from tomato and spider flower *Cleome hassleriana* had no detectable effect, indicating that the induction of crop resistance is dependent on banker plant species. Moreover, *T. urticae* infestation of banker plants consistently suppressed mite oviposition on neighboring tomato plants across all banker plant species tested. These findings suggest that banker plants can exert previously unrecognized bottom-up effects by modulating crop resistance-related responses through airborne cues. Therefore, selecting banker plant species that emit effective airborne cues may strengthen crop protection and stabilize biological control performance in sustainable IPM strategies.

## 1. Introduction

Non-crop plants are widely incorporated into agricultural systems to enhance the effectiveness of biological control agents [1,2]. Among these, banker plants facilitate the early establishment of biological control agent populations by providing alternative food sources, including pest or non-pest herbivores, in both field and greenhouse environments [3,4,5,6]. The characteristics of leaves, stems, and flowers can influence the survival and reproduction of biological control agents either positively or negatively. In particular, pollen-producing plants are employed as banker plants to improve the performance of predatory bugs and mites [7,8,9]. Moreover, certain predatory mirid bugs, such as *Nesidiocoris tenuis* (Reuter) (Hemiptera: Miridae), can develop and reproduce on plant tissue alone [10,11,12,13,14]. Sesame *Sesamum indicum* L. and spider flower *Cleome hassleriana* Chodat are commonly used as banker plants for *N. tenuis* due to their zoophytophagous characteristics [15,16].

Plants deploy a suite of inducible direct and indirect responses that reduce herbivore performance and mediate multitrophic interactions [17]. Direct defenses include mechanical protection on the plant surface or toxin and anti-digestive protein production that impair herbivore fitness [18]. Indirect defenses involve the emission of a blend of herbivory-induced plant volatiles (HIPVs), which attract natural enemies, provide food and shelter, and thereby enhance biological control [19,20]. HIPVs also influence the behavior of neighboring plants, herbivores, and natural enemies across tritrophic levels [20,21,22]. Exposure to HIPVs can prime or amplify direct and indirect defense pathways in neighboring plants, thereby reducing their susceptibility to future herbivory [23,24,25].

Zoophytophagous biological control agents, including the hemipteran families Miridae, Anthocoridae, and Pentatomidae and the mite family Phytoseiidae [26,27], can also elicit plant-feeding responses. *Nesidiocoris tenuis*, similar to many zoophytophagous mirid bugs, punctures plant tissues, triggering direct defenses that reduce the performance of pest herbivores, such as the two-spotted spider mite *Tetranychus urticae* Koch (Acari: Tetranychidae) and South American tomato moth *Tuta absoluta* (Meyrick) (Lepidoptera: Gelechiidae) [28,29,30]. Feeding by *N. tenuis* has been shown to induce HIPV emission, thereby potentially influencing herbivore and natural enemy behavior and performance [31,32,33,34]. These *N. tenuis*-induced HIPVs (Nt-HIPVs) can prime or enhance direct defenses in neighboring intact crops, reducing fecundity of the Kanzawa spider mite *Tetranychus kanzawai* Kishida (Acari: Tetranychidae) and suppressing weight gain of the tobacco cutworm *Spodoptera litura* (Fabricius) (Lepidoptera: Noctuidae) larvae on eggplant [35]. Synthetic Nt-HIPV has similarly been shown to induce direct defenses in intact tomato plants, rendering them repellent to the sweet potato whitefly *Bemisia tabaci* (Gennadius) (Hemiptera: Aleyrodidae), *T. absoluta*, and western flower thrips *Frankliniella occidentalis* Pergande (Thysanoptera: Thripidae), while reducing the performance of *T. urticae* and *T. absoluta* under commercial greenhouse conditions [36,37].

Banker plants infested with *N. tenuis* release airborne cues that attract conspecifics [33,34]. Although banker plant systems expose neighboring crops to these airborne cues prior to pest outbreaks, the potential bottom-up effects from banker plants remain poorly understood. In this study, we hypothesized that such airborne cues from banker plants infested with *N. tenuis* would modulate defensive responses in neighboring, intact tomato plants. To test this, we conducted two experiments to assess whether airborne communication initiated by (1) *N. tenuis* infestation or (2) *T. urticae* infestation on banker plants affects the survival and reproduction of *T. urticae* on neighboring tomato plants. Despite the widespread use of banker plant systems to promote biological control, it remains unclear whether these systems also confer bottom-up benefits by enhancing or altering crop resistance. To address this gap, we tested the hypothesis that banker plant-derived airborne cues affect the performance of the two-spotted spider mite on neighboring tomato plants.

## 2. Results

### 2.1. Effects of N. tenuis Herbivory on Banker Plants on T. urticae Oviposition in Neighboring Tomato Plants

**Figure 1:**
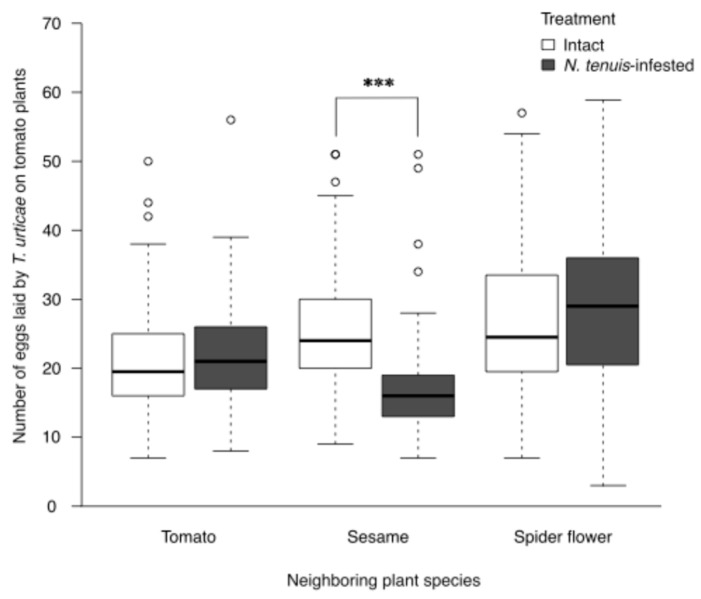
Effects of *Nesidiocoris tenuis*-herbivory on banker plants on oviposition of *Tetranychus urticae* in neighboring tomato plants. Number of eggs laid by *T. urticae* on neighboring tomato plants exposed to intact or *N. tenuis*-infested plants (tomato, sesame, and spider flower). GLMM analysis revealed a significant interaction between treatment and plant species (*p* = 0.0002). Post hoc comparisons indicated that *N. tenuis*-infested sesame significantly reduced the number of eggs (*p* < 0.0009), whereas no significant effects were observed for tomato (*p* = 0.6175) or spider flower (*p* = 0.1345). Sample sizes were n = 60 (intact tomato), 58 (infested tomato), 45 (intact sesame), 45 (infested sesame), 60 (intact spider flower), and 60 (infested spider flower). Significant differences are indicated by “***” (*p* < 0.001).

To evaluate the effect of infestation status (hereafter referred to as ‘treatment’: intact, infested) and banker plant species, we compared the oviposition and survival of *T. urticae* on tomato plants. *Nesidiocoris tenuis*-infested plants significantly affected the number of eggs laid by *T. urticae* on neighboring tomato plants, and the magnitude of the effect varied depending on the plant species (treatment: χ^2^ = 0.7415, df = 1, *p* = 0.3892; species: χ^2^ = 12.6481, df = 2, *p* = 0.0018; treatment × plant: χ^2^ = 17.5481, df = 2, *p* = 0.0002, GLMM; Figure 1). Specifically, *N. tenuis*-infested sesame plants significantly reduced the number of eggs compared with intact plants (χ^2^ = 11.059, df = 1, *p* = 0.0009, GLMM; Figure 1). Conversely, *N. tenuis*-infested tomato and spider flower plants had no significant effect (tomato: χ^2^ = 0.2494, df = 1, *p* = 0.6175; spider flower: χ^2^ = 2.2401, df = 1, *p* = 0.1345, GLMM; Figure 1).

### 2.2. Effects of N. tenuis Herbivory on Banker Plants on T. urticae Survival in Neighboring Tomato Plants

**Figure 2:**
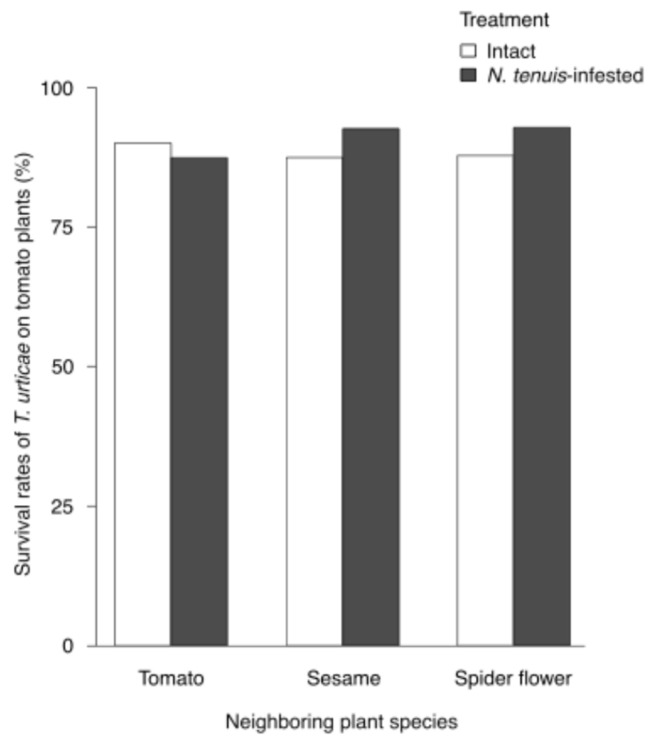
Effects of *Nesidiocoris tenuis* herbivory on banker plants on *Tetranychus urticae* survival in neighboring tomato plants. Survival rates of *T. urticae* on neighboring tomato plants exposed to intact or *N. tenuis*-infested plants (tomato, sesame, and spider flower). GLMM analysis indicated no significant effects of treatment (*p* = 0.2627), plant species (*p* = 0.9538), or their interaction (*p* = 0.1535). Sample sizes were n = 60 (intact tomato), 58 (infested tomato), 45 (intact sesame), 45 (infested sesame), 60 (intact spider flower), and 60 (infested spider flower).

*Nesidiocoris tenuis*-infested plants did not significantly affect the survival rates of *T. urticae* on neighboring tomato plants (treatment: χ^2^ = 1.2545, df = 1, *p* = 0.2627; plant: χ^2^ = 0.0947, df = 2, *p* = 0.9538; treatment × plant: χ^2^ = 3.7484, df = 2, *p* = 0.1535, GLMM; Figure 2).

### 2.3. Effects of T. urticae Herbivory on Banker Plants on T. urticae Oviposition in Neighboring Tomato Plants

**Figure 3:**
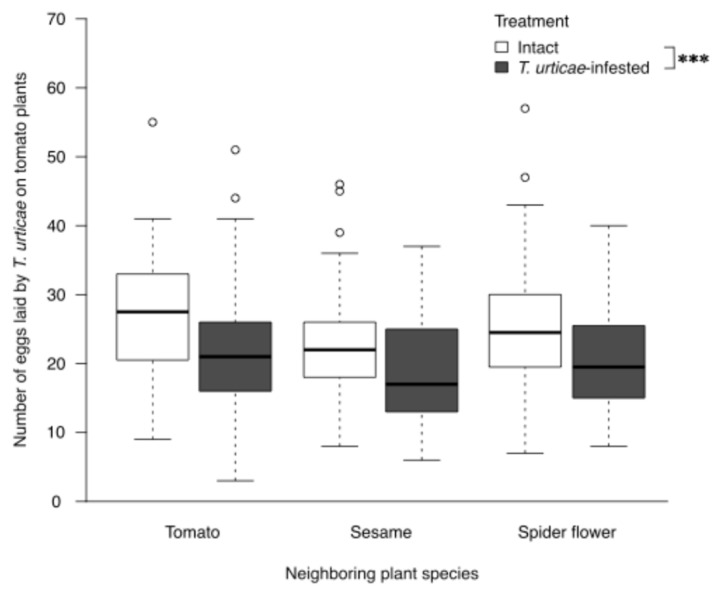
Effects of *Tetranychus urticae* herbivory on banker plants on oviposition of *T. urticae* in neighboring tomato plants. Number of eggs laid by *T. urticae* on neighboring tomato plants exposed to intact or *T. urticae*-infested plants (tomato, sesame, and spider flower). GLMM analysis showed a significant main effect of treatment (*p* < 0.0001), indicating that *T. urticae* herbivory significantly reduced the number of eggs, regardless of neighboring plant species. The interaction between treatment and plant species was not significant (*p* = 0.5211). Sample sizes were n = 60 (intact tomato), 60 (infested tomato), 45 (intact sesame), 45 (infested sesame), 60 (intact spider flower), and 60 (infested spider flower). Significant differences are indicated by “***” (*p* < 0.001).

*Tetranychus urticae*-infested plants significantly reduced the number of eggs laid by *T. urticae* on neighboring tomato plants (χ^2^ = 20.9696, df = 1, *p* < 0.0001, GLMM; Figure 3). The effects of plant species (χ^2^ = 1.3035, df = 2, *p* = 0.5211; Figure 3) and treatment × plant interaction (χ^2^ = 0.5022, df = 2, *p* = 0.7780, GLMM; Figure 3) were not significant.

### 2.4. Effects of T. urticae Herbivory on Banker Plants on T. urticae Survival in Neighboring Tomato Plants

**Figure 4:**
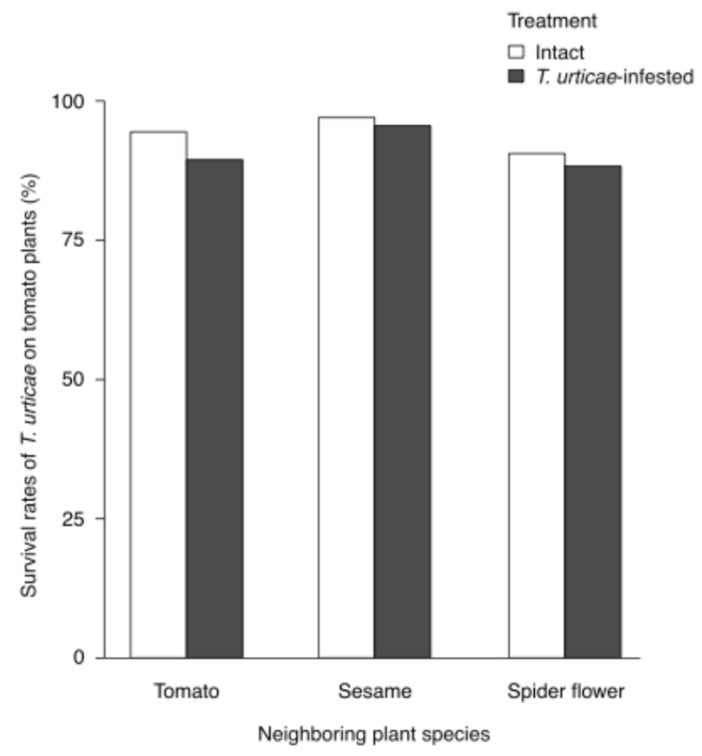
Effects of *Tetranychus urticae* herbivory on banker plants on *T. urticae* survival in neighboring tomato plants. Survival rates of *T. urticae* on neighboring tomato plants exposed to intact or *T. urticae-*infested plants (tomato, sesame, and spider flower). GLMM analysis indicated no significant effects of treatment (*p* = 0.0890), plant species (*p* = 0.0713), or their interaction (*p* = 0.6998). Sample sizes were n = 60 (intact tomato), 60 (infested tomato), 45 (intact sesame), 45 (infested sesame), 60 (intact spider flower), and 60 (infested spider flower).

*Tetranychus urticae*-infested plants did not significantly affect *T*. *urticae* survival on neighboring tomato plants (treatment: χ^2^ = 2.8925, df = 1, *p* = 0.0890; plant: χ^2^ = 5.2814, df = 2, *p* = 0.0713; treatment × plant: χ^2^ = 0.7140, df = 2, *p* = 0.6998, GLMM; Figure 4).

## 3. Discussion

This study suggests that airborne cues emitted from banker plants can alter herbivore performance on neighboring crop plants by modulating their resistance-related responses. While the underlying physiological and molecular mechanisms warrant further investigation, these findings highlight a potential bottom-up pathway in banker plant systems that complements their established role in supporting biological control agents. Importantly, the number of *T. urticae* eggs laid on crops neighboring plants infested by biological control agents was significantly influenced by the interaction between arthropods and banker plant species, underscoring the critical importance of selecting appropriate plant species.

The number of eggs laid by *T. urticae* on tomato plants adjacent to *N. tenuis*-infested plants was significantly influenced by the interaction between the treatment and plant species. This suggests that proximity to *N. tenuis*-infested plants affects the resistance-related responses of neighboring tomato plants, depending on plant species. Specifically, the presence of *N. tenuis*-infested sesame plants significantly decreased the number of eggs laid by *T. urticae* compared with that of intact plants. However, tomato and spider flower plants adjacent to *N. tenuis*-infested plants did not influence the number of eggs on the neighboring tomato plants. These differences suggest that the response of tomato plants to *N. tenuis*-infested plants varies among banker plant species. Furthermore, the observed reduction in egg numbers might be attributed not only to these bottom-up resistance-related responses but also to additional effects, such as potential repellent properties of the airborne cues or their adsorption onto the recipient plant surfaces, which could directly interfere with herbivore settlement. Regarding *T. urticae* infestation, the treatment significantly reduced the number of eggs laid by *T. urticae* on tomato plants neighboring *T. urticae*-infested plants, regardless of the plant species. The lack of a significant interaction between the treatment and plant species indicates that proximity to *T. urticae*-infested plants consistently affected *T. urticae* performance, potentially by enhancing the resistance-related responses of neighboring tomato plants.

In a previous study, eggplants placed in proximity to *N. tenuis*-infested conspecific plants exhibited jasmonic acid priming and enhanced resistance, which reduced the fecundity of *T. kanzawai* [35]. Similarly, tomato plants exposed to a major volatile compound [(*Z*)-3-hexenyl propanoate] from conspecific HIPVs reduced subsequent phytophagous infestations by *T. urticae* [36]. The observed reduction in *T. urticae* performance of tomato plants adjacent to *N. tenuis*-infested sesame plants aligns with the previous findings. We hypothesize that airborne cues from sesame plants may trigger resistance-related responses in tomato; however, further physiological and molecular analyses are required to confirm the underlying mechanisms.

From a practical perspective, the effectiveness of these effects mediated by airborne cues in real agricultural systems warrants careful evaluation. Environmental factors, including air currents and temperature fluctuations, may affect the diffusion and perception of these cues differently than in laboratory settings. Furthermore, some banker plant species may inadvertently serve as alternative hosts for pest populations, potentially complicating pest management if biological control agents fail to adequately suppress these pests. However, our findings suggest that bottom-up modulation of crop resistance may mitigate these risks by reinforcing inherent plant resistance. Balancing these complex interactions is key to optimizing the use of banker plants.

Traditionally, the benefits of banker plants have been mainly attributed to their top-down effects, particularly pest suppression by biological control agents. Our findings suggest an additional bottom-up pathway, in which airborne cues emanating from banker plants infested with biological control agents can modulate the resistance-related responses of neighboring crops. The direction and magnitude of these effects vary among banker plant species, underscoring the importance of selecting plant species that provide beneficial airborne cues. Incorporating bottom-up effects in banker plant design may enhance the stability of biological control and promote more resilient and sustainable integrated pest management strategies.

## 4. Materials and Methods

### 4.1. Plants

Tomato *Solanum lycopersicum* L. (cv. Momotaro York), *S. indicum* (cv. Kanto No. 1), and *C. hassleriana* (cv. Color Fountain) were individually cultivated in plastic pots (diameter, 7.5 cm; height, 6.5 cm) in a greenhouse (25 ± 3 °C, 90 ± 4% RH, and natural day length). Kidney bean plants *Phaseolus vulgaris* L. (cv. Nagauzura) were individually cultivated in a climate-controlled room (25 ± 3 °C, 20 ± 10% RH, 16L:8D photoperiod). Each potted plant was approximately 15 cm in height.

### 4.2. Arthropods

*Nesidiocoris tenuis* was collected from sesame plants in Kasugai, Aichi, Japan, in September 2017. They were reared in a controlled growth chamber (25 ± 1 °C, 90 ± 4% RH, 16L:8D photoperiod), fed with eggs of the Mediterranean flour moth *Ephestia kuehniella* Zeller (Lepidoptera: Pyralidae), and provided with leaves from the jelly bean plant *Sedum rubrotinctum* R.T. Clausen or jade plant *Crassula ovata* (Mill.) Druce as oviposition substrates and water sources. Third-instar nymphs of *N. tenuis* were used in experiments to evaluate the effects of the feeding stimulus.

*Tetranychus urticae* was reared in a growth chamber under identical conditions using square leaf pieces (5 cm × 5 cm) of kidney bean primary leaves, which were placed on water-saturated cotton wool in plastic Petri dishes (diameter, 90 mm; height, 20 mm).

### 4.3. Responses of Tomato Plants Adjacent to N. tenuis-Infested Banker Plants

Acrylic boxes (35 cm × 25 cm × 30 cm) with windows on two sides (10 cm × 15 cm) and a back (35 cm × 30 cm), covered with nylon mesh (opening 181 µm), were used. The two boxes were interconnected in a back-to-back configuration to allow airborne cues to pass through. Air circulation between the boxes occurred passively, depending on natural diffusion via the nylon mesh windows. Three tomato plants were placed in one box as recipient plants, while three plants of the same species were placed in the other box as airborne cues emanating from plants. Three plant species were tested: tomato, sesame, and spider flower. Thirty third-instar *N. tenuis* nymphs were introduced to the plants. Tomato plants served as a control by acting as neighboring uninfested plants, following the same procedure described above. The two connected boxes were placed in a greenhouse (25 ± 3 °C, 90 ± 4% RH, natural day length). The positions of the boxes within the greenhouse were randomized within each replicate to minimize potential confounding effects from microclimate. The greenhouse experiments were conducted with four replicates for tomato and spider flower plants and three replicates for sesame plants; the lower number of replicates for sesame was due to the limited availability of uniform plants during the experimental period.

After seven days, three to five square leaf pieces (1.5 cm × 1.5 cm) were cut from each tomato plant that had been placed in proximity to *N. tenuis*-infested or intact plants. Each leaf piece was placed on water-saturated cotton wool in a plastic Petri dish. Three *T. urticae* females were placed on each leaf piece. The dishes were kept in a growth chamber (25 ± 1 °C, 90 ± 4% RH, 16L:8D photoperiod). After three days, the number of surviving *T. urticae* and the number of eggs laid by *T. urticae* were counted. Thirteen to fifteen leaf pieces were collected from the three plants per replicate. The total number of leaf pieces analyzed was as follows: 60 each for intact tomato, intact spider flower, and infested spider flower; 58 for infested tomato; and 45 each for intact and infested sesame.

### 4.4. Responses of Tomato Plants Neighboring T. urticae-Infested Banker Plants

The materials and methods used in this experiment were the same as those used in the *N. tenuis*-infested experiment, except for the treatment of infested plants. A total of 150 *T. urticae* females were introduced to the plants. *N. tenuis* and *T. urticae* experiments were conducted alternately to account for seasonal variations in day length and environmental conditions. Fifteen leaf pieces were collected from the three recipient plants per replicate. The total number of leaf pieces analyzed was as follows: 60 each for intact tomato, infested tomato, intact spider flower, and infested spider flower, and 45 each for intact and infested sesame.

### 4.5. Statistical Analysis

We employed a generalized linear mixed model (GLMM) in R version 4.1.1 (R Foundation for Statistical Computing, Vienna, Austria) to analyze the effects of treatment (intact, infested), plant species (tomato, sesame, spider flower), and their interaction [37]. The GLMM was performed using the glmer function from the lme4 package version 1.1–27.1 [38] to examine the effects of the number of eggs laid by *T. urticae*, modeled with a Poisson distribution, and survival rates of *T. urticae*, modeled with a binomial distribution. We included treatment (intact, infested), plant species (tomato, sesame, spider flower), and their interaction as fixed effects. The date of the experiment and individual plant identity were included as random effects to account for the hierarchical structure of the data (leaf pieces nested within plants) and environmental variations. The significance of each model was evaluated through the Type II Wald chi-square test with the Anova function from the car package version 3.1.2 (Sage, Thousand Oaks, CA, USA) [39] in R. When the interaction significantly affected the number of the eggs or survival rates, we analyzed the effects of treatment on the number of eggs or survival rates for each type of plant using a GLMM through the glmer function in the lme4 package in R. The significance of each model was evaluated through the likelihood ratio test with the anova function in R.

## Data Availability

All data are available at https://doi.org/10.57723/kds590431.
